# Beyond the Milky Pleural Fluid: A 14-Year Study on Non-Traumatic Chylothorax From a Tertiary Care Hospital in India

**DOI:** 10.7759/cureus.80381

**Published:** 2025-03-11

**Authors:** Aneef K Basha, Anupam Prakash, Kanishka K Singh, Deepak Prajapat, Dhruv Talwar, Deepak Talwar

**Affiliations:** 1 Pulmonary and Critical Care Medicine, Metro Centre for Respiratory Disease, Metro Multispecialty and Heart Institute, Noida, IND; 2 Pulmonary Medicine, All India Institute of Medical Sciences, Patna, Patna, IND

**Keywords:** chylous effusion, complicated pleural effusion, effusion, recurrent effusion, undiagnosed pleural effusion

## Abstract

Chylothorax is an uncommon type of pleural effusion caused by the accumulation of chyle in the pleural space. It represents 2% to 3% of pleural effusions and can be classified into two categories: traumatic and non-traumatic. Chylothorax may carry high morbidity and mortality, especially when associated with trauma or malignancies. We present five cases of non-traumatic chylothorax, confirmed by pleural fluid analysis, with different etiologies seen at the Metro Center for Respiratory Diseases, Noida, from 2010 to 2024. These cases represent the wide spectrum of conditions associated with this unusual finding.

## Introduction

Chylothorax is an uncommon type of pleural effusion caused by the accumulation of chyle in the pleural space. It can be classified into four categories: traumatic, malignant, idiopathic, or miscellaneous [1]. Chylothorax may be difficult to manage, especially when associated with trauma or malignancies [2]. Malignancy accounts for one-third of non-traumatic cases, and the spectrum of non-malignant causes (congenital, infectious, etc.) is diverse [3].

The diagnosis of chylothorax relies on the analysis of the pleural fluid. Chyle consists of chylomicrons, triglycerides (TG), cholesterol, fat-soluble vitamins, lymphocytes, immunoglobulins, and enzymes [4]. Although the fluid is expected to be milky white in color, less than 50% of cases have this feature, which may obscure the diagnosis. Lactate dehydrogenase and protein content analysis can assist in narrowing the differential diagnosis. Lymphocytic-predominant, protein-discordant exudative effusion is more common than transudative. Chylothorax is diagnosed when a pleural effusion has a TG concentration greater than 110 mg/dL. The presence of chylomicrons is the gold standard for diagnosis but is only necessary when the TG concentration is above 110 mg/dL [3].

There is limited data on the most suitable approach to manage chylothoraces, and treatment often depends on the underlying cause. In general, conservative treatment is tried first, usually for a limited time, before considering more invasive measures. A multidisciplinary approach is recommended with close collaboration among respiratory physicians, thoracic surgeons, oncologists, interventional radiologists, and dietitians.

## Case presentation

Case 1

A 63-year-old woman was referred to our unit for recurrent pleural effusion of unknown cause. She had a history of on-and-off right upper limb swelling for 40 years and multiple thoracenteses. She was treated with antitubercular therapy (ATT) on two occasions, and the last pleural tapping in June 2012 was reportedly hemorrhagic. In view of recurrent pleural effusion, video-assisted thoracoscopic surgery (VATS) biopsy with tube thoracostomy was done. The pleural fluid drained was hemorrhagic and blood-stained. On pleural fluid evaluation, triglycerides and chylomicrons were positive, confirming chylothorax.

Detailed evaluation, including positron emission tomography-computed tomography (PET-CT) and lymphoscintigraphy, did not reveal any malignancy or lymphatic abnormality. VATS pleural biopsy revealed non-specific chronic inflammation. The patient was kept on a restricted-fat diet with medium-chain triglycerides. Intercostal drain (ICD) reduced initially but increased again. In view of persistent chylothorax, a pleuroperitoneal shunt was planned. After consultation with cardiothoracic and vascular surgery (CTVS), the ICD was removed, and the patient was discharged in stable condition.

Case 2

A 30-year-old man with tubercular meningitis (on ATT and steroids) and seizure disorder (on phenytoin) was referred for left-sided undiagnosed pleural effusion. The patient underwent a medical thoracoscopy and biopsy, and 750 mL of turbid reddish-yellow fluid was aspirated for workup. Pleural fluid characteristics confirmed chylothorax. Thoracoscopic-guided pleural biopsy revealed non-specific pleuritis. Lymphoscintigraphy showed normal lymphatic drainage. The patient was managed conservatively with an oral medium-chain triglyceride diet.

Case 3

**Figure 1:**
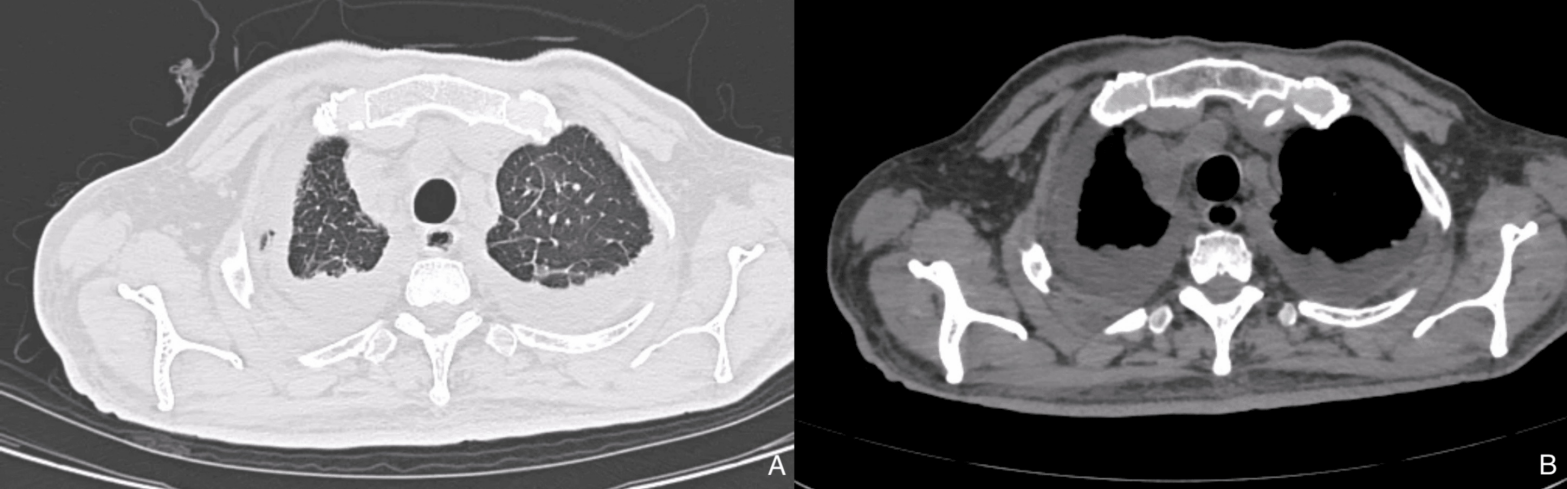
HRCT chest shows moderate bilateral pleural effusion and areas of interlobular septal thickening HRCT: High-resolution computed tomography, A: Lung window, B: Mediastinal window

A 60-year-old male presented with a cough and fever for 4-5 days. He was a known case of chronic myeloid leukemia. High-resolution computed tomography (HRCT) chest (Figure 1) showed moderate bilateral pleural effusion and areas of interlobular septal thickening and bronchial wall thickening. Pleural tapping was done from the right side, and the workup was negative for pyogenic culture and malignant cells. Elevated pleural TG levels confirmed chylothorax. The patient was managed conservatively with an oral medium-chain triglyceride diet.

Case 4

**Figure 2:**
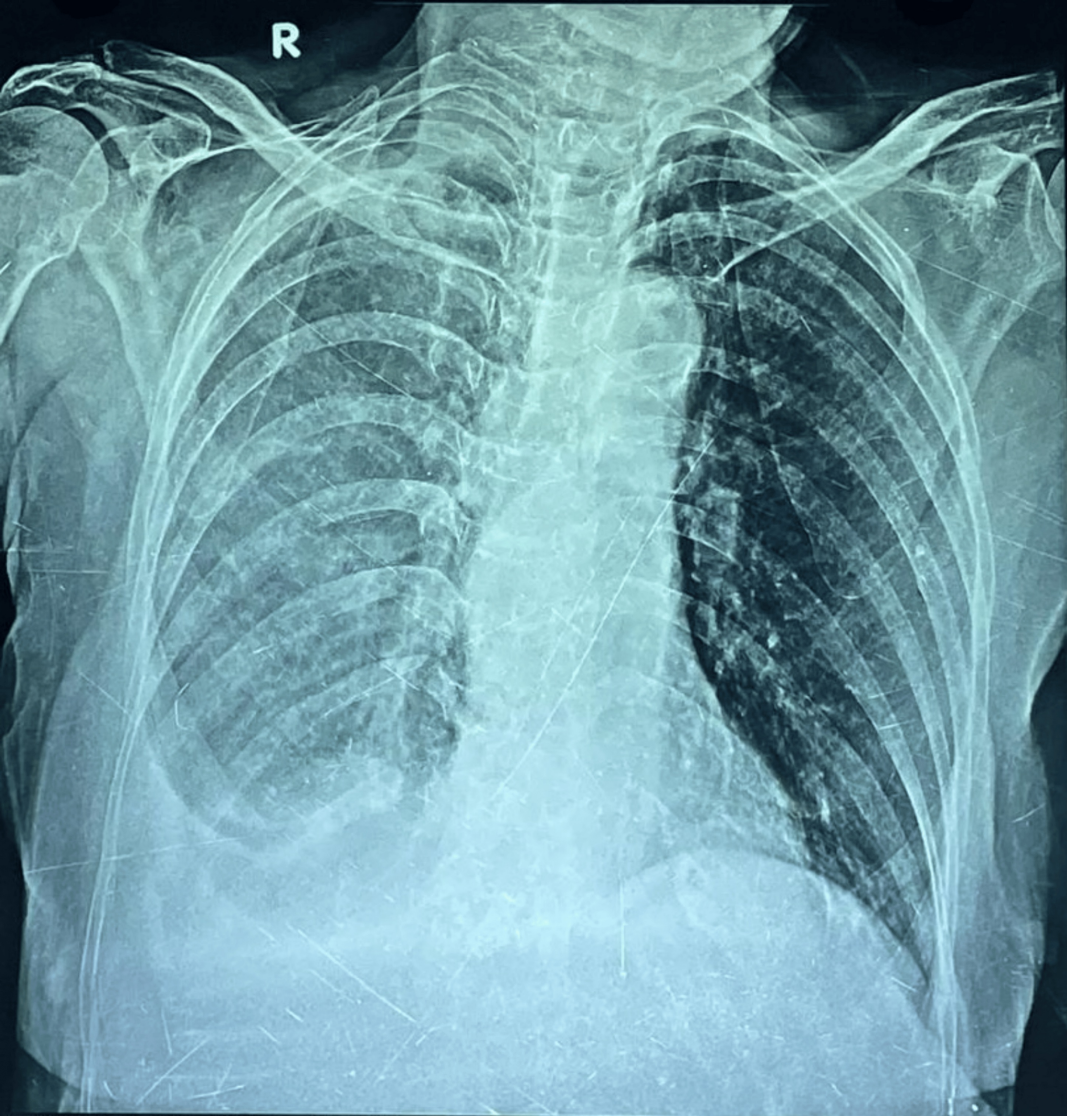
Chest X-ray shows right-sided pleural effusion

A 63-year-old female with a history of yellow nails and lymphadenopathy for 15 years presented with shortness of breath for one month. A routine chest X-ray (Figure 2) revealed a right-sided pleural effusion. Pleural tapping and workup were negative for pyogenic culture and malignant cells. Elevated pleural TG levels confirmed chylothorax. Thoracoscopy followed by talc pleurodesis was performed. The patient was managed conservatively with an oral medium-chain triglyceride diet.

Case 5

**Figure 3:**
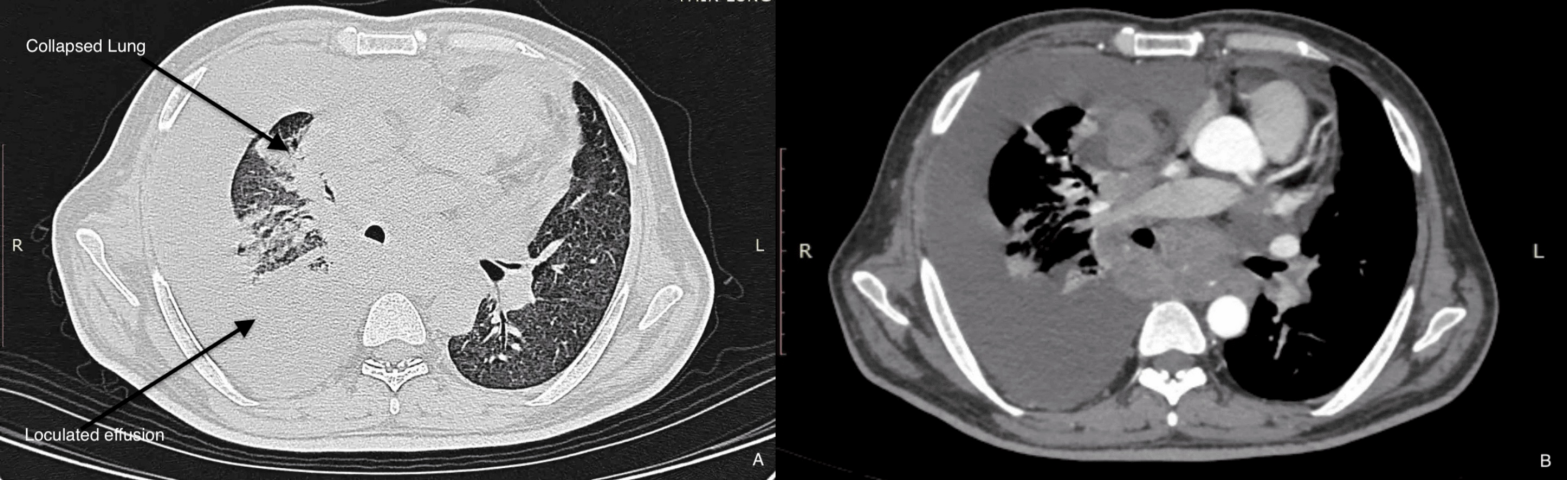
HRCT showing right moderate loculated pleural effusion with partial lung collapse HRCT: High-resolution computed tomography, A: Lung window, B: Mediastinal window

**Figure 4:**
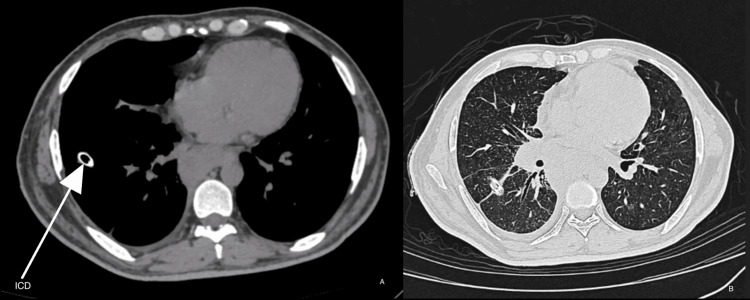
HRCT chest showing ICD in situ HRCT: High-resolution computed tomography, ICD: Intercostal drain, A: Mediastinal Window, B: Lung Window

**Figure 5:**
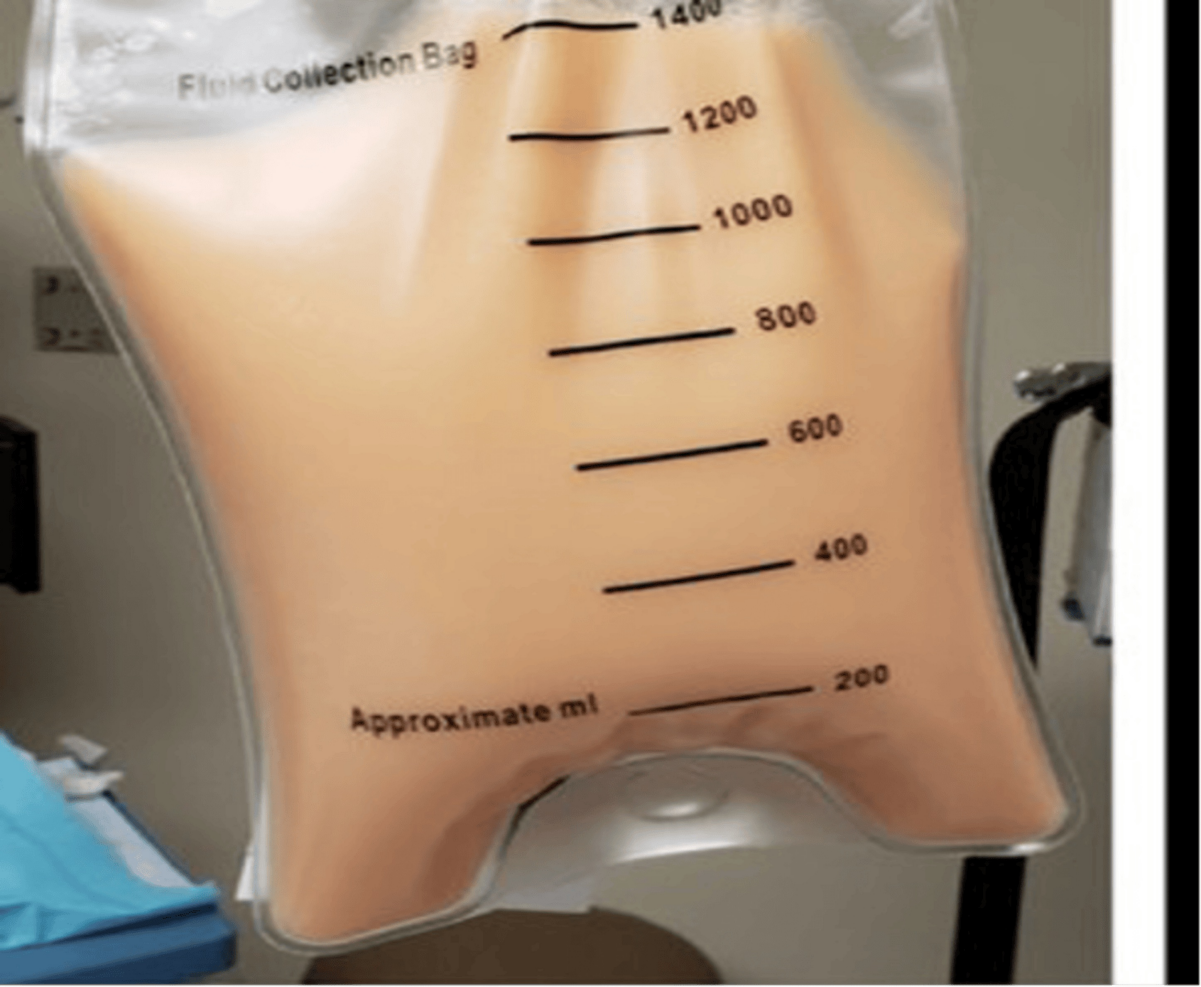
Milky fluid in ICD bag ICD: Intercostal drain

A 34-year-old male was transferred for management of undiagnosed pleural effusion. He presented with cough, shortness of breath, and loss of appetite for three days. He was a known case of disseminated tuberculosis (Sputum CBNAAT - MTB detected and Cervical LN biopsy - necrotic lymph nodes) (on ATT for two months). HRCT showed right moderate loculated pleural effusion with partial lung collapse (Figure 3). ICD was placed (Figure 4), and pleural fluid analysis showed elevated TG levels and chylomicrons (Figure 5). PET-CT ruled out neoplastic etiology, and lymphoscintigraphy showed no evidence of thoracic duct leakage. The patient was managed conservatively and discharged with an ICD in situ.

## Discussion

Chylothorax develops when a disruption or blockage of the thoracic duct leads to the leakage of chyle into the pleural space. Table 1 summarizes the critical findings of each case in the case series. Its presentation is similar to other types of pleural effusion, commonly with cough and shortness of breath. Fever and pleurisy are uncommon due to the nonirritative nature of chyle [4].

**Table 1:** Clinical presentation, etiological, and diagnostic and therapeutic approaches of all cases TG: Triglycerides, PET-CT: Positron emission tomography computed tomography, HRCT: High-resolution computed tomography, ICD: Intercostal drain

Case Number	Etiology	Patient Details	Diagnostic Modality Used	Diagnostic Findings	Treatment Approach	Outcome
Case 1	Non-traumatic	63-year-old female with recurrent pleural effusion, intermittent upper limb swelling, history of trauma 20 years ago, and multiple thoracenteses	PET-CT, lymphoscintigraphy, pleural fluid analysis	No lymphatic abnormalities; TG-870 mg/dl	Restricted fat diet, pleuroperitoneal shunt	Stable, discharged
Case 2	Non-traumatic	30-year-old male with tubercular meningitis, seizure disorder, and turbid reddish-yellow pleural effusion	Medical thoracoscopy, biopsy, lymphoscintigraphy	TG-169 mg/dl, nonspecific pleuritis	Medium-chain triglyceride diet	Stable, discharged
Case 3	Non-traumatic	60-year-old male with chronic myeloid leukemia, cough, and fever for 4–5 days	HRCT chest, pleural fluid analysis	TG - 244 mg /dl, no malignant cells	Medium-chain triglyceride diet	Stable, discharged
Case 4	Non-traumatic	63-year-old female with yellow nails, lymphadenopathy for 15 years, and shortness of breath for 1 month	Routine chest X-ray, thoracoscopy	TG- 210 mg/dl, yellow nail syndrome suspected	Pleurodesis with talc	Stable, discharged
Case 5	Non-traumatic	34-year-old male with disseminated tuberculosis, cough, loss of appetite, and loculated pleural effusion	HRCT chest, PET-CT, lymphoscintigraphy	Loculated effusion, TG-145.7mg/dl	Conservative management	Stable, discharged with ICD

In nontraumatic cases, fluid accumulation is slow, and the presentation is often indistinguishable from other causes of pleural effusion. Conservative measures and treatment of the underlying disease remain the primary approach. However, these measures are not always effective, necessitating surgical interventions such as talc pleurodesis, which was performed in three cases in our study.

Along with pleural fluid analysis, multiple diagnostic modalities can aid in the evaluation of chylothorax. Lymphoscintigraphy is helpful for both diagnosis and localization of leakage sites that may require surgical intervention [5].

Although lymphoscintigraphy has been reported to have 88% sensitivity and 100% specificity in some case reports, our case series (three cases) did not reveal significant thoracic duct injury [5]. Lipoprotein electrophoresis of pleural fluid demonstrating chylomicrons can confirm the diagnosis of chylothorax [6,7]. Lymphoma accounts for approximately 70% of malignant causes of chylothorax, typically due to direct invasion of the lymphatic system or external compression by a tumor. Magnetic resonance lymphangiography can provide insights into the mechanism of chylothorax caused by neoplasms, including the location of obstruction [3,4].

However, non-neoplastic etiologies causing chylothorax are rare, with only a few reported cases [8-10]. In our case series, one case (Case 5) of chylothorax was attributed to tuberculosis, which is an uncommon manifestation. It typically occurs due to direct involvement of the thoracic duct by tuberculous lymphadenitis or inflammation, leading to duct obstruction or rupture. More frequently, tuberculosis (TB) is associated with pseudochylothorax (cholesterol pleurisy), resulting from long-standing pleural effusions where cholesterol accumulates, giving the fluid a milky appearance [11]. Additionally, a paradoxical reaction to antitubercular therapy (ATT) can, in rare cases, lead to chylothorax. This occurs due to an exaggerated immune response following mycobacterial antigen release during treatment, triggering inflammation and potential disruption of the thoracic duct or its tributaries. However, documented cases of chylothorax as a paradoxical reaction to ATT are scarce, underscoring its rarity [12].

Yellow nail syndrome (YNS) (Case 4) is another rare cause of chylothorax. Characterized by yellow, thickened nails, lymphedema, and recurrent respiratory symptoms, YNS can lead to pleural effusions, including chylothorax. The proposed mechanism involves structural abnormalities in the lymphatic system, such as hypoplasia or dilation of lymphatic vessels, leading to chyle leakage into the pleural space. This condition highlights the role of intrinsic lymphatic dysfunction in chylothorax pathogenesis [13].

When comparing pleural fluid analyses among our cases, variability in triglyceride [TG] levels was observed. Nevertheless, the TG levels always exceeded the diagnostic threshold of 110 mg/dL [1.24 mmol/L]. The pleural fluid was consistently exudative in nature. It was milky in appearance in only one case, while the rest were non-milky. This variability aligns with previous observations, where the characteristic milky appearance is seen in only half of the cases, with serosanguineous, bloody, or serous fluid also being described (Table 2). Milky chylothorax occurs due to the high fat content in the chyle, which gives it a characteristic milky appearance, especially after eating when dietary fats are absorbed and transported via the thoracic duct. In contrast, non-milky chylothorax appears clear or slightly cloudy and can result from fasting, a low-fat diet, or a small chyle leak, leading to reduced fat content in the pleural fluid. While a milky appearance suggests chylothorax, definitive diagnosis requires pleural fluid analysis, specifically elevated triglyceride levels and the presence of chylomicrons [3,14].

**Table 2:** Pleural fluid analysis of cases ADA: Adenosine deaminase and LDH: Lactate dehydrogenase

Pleural fluid Parameters	CASE 1	CASE 2	CASE 3	CASE 4	CASE 5
Color	Hemorrhagic	Reddish Yellow	Milky	Yellow	Milky
Glucose	149 mg/dl	105mg/dl	60.5 mg/dl	80 mg/dl	116 mg/dl
Protein	4.5 mg/dl	3.8 mg/dl	4.73 mg/dl	4.5 mg/dl	6.24 mg/dl
ADA	21.8 u/l	18.7 u/l	15 u/l	36 u/l	39 u/l
LDH	96 u/l	88.8 u/l	2684 u/l	476 u/l	165 u/l
Triglycerides	870 mg/dl	169 mg/dl	244 mg /dl	210 mg/dl	145.7mg/dl
Chylomicrons	20 mg/dl	39.9	82 mg/dl	90 mg/dl	54.2 mg/dl

The management of chylothorax requires a multidisciplinary approach involving dietary, medical, and surgical interventions. A low-fat diet with medium-chain triglycerides (MCT) is believed to reduce chyle production, while total parenteral nutrition (TPN) is suggested as the next step if dietary measures fail [15]. Somatostatin analogs have shown promise in managing chylothorax, especially in pediatric patients, as evidenced by small trials [16]. Additionally, midodrine, an A1 agonist, has demonstrated effectiveness in treating refractory chylothorax, although data are primarily derived from case reports [17].

## Conclusions

Non-traumatic chylothorax is a rare condition with diverse causes requiring accurate diagnosis and a tailored, multidisciplinary approach. Our cases highlight the variability in presentation and the need for individualized management strategies for effective outcomes.
